# Community voices from modern maternal waiting home users in Zambia

**DOI:** 10.3389/fgwh.2024.1444611

**Published:** 2024-08-15

**Authors:** Melvin Kunda Mwansa, Kafiswe Chimpinde, Mergan Naidoo

**Affiliations:** ^1^ Discipline of Public Health Medicine, School of Nursing and Public Health, University of KwaZulu Natal, Durban, South Africa; ^2^ Department of Demography, Population Sciences, Monitoring & Evaluation, School of Humanities & Social Sciences, University of Zambia, Lusaka, Zambia; ^3^ Discipline of Family Medicine, School of Nursing and Public Health, University of KwaZulu Natal, Durban, South Africa

**Keywords:** modern maternal waiting homes, health facilities, users, non-users, utilisation

## Abstract

**Objective:**

This study aimed to understand community voices on factors influencing utilisation of MMWHs in Zambia.

**Methods:**

The study employed a mixed method study design in four selected health facilities across Zambia districts between January 2021 and December 2022. Purposeful sampling was used to select study participants using MMWH registers as the sampling frame for mothers who had utilised MMWHs and their spouses. Sampling of participants through face-to-face, in-depth interviews (IDIs) and focus group discussions (FGDs) was conducted to saturation in all targeted health facilities Data was transcribed verbatim and analysed thematically.

**Results:**

Results found that the comfortable state of the MMWHs, long distances to health facilities, fear of maternal complications, availability and positive attitudes of specialized health personnel, and the information on childcare provided were major factors. Spouses supported their partners using MMWHs due to the quality of care and the availability of modern equipment and furniture. However, some spouses indicated that utilisation of MMWHs has a bearing on a household's financial resources.

**Conclusion:**

MMWHs are a pivotal intervention in improving maternal outcomes. All health facilities had no maternal and newborn complications or deaths over the study period.

## Introduction

The World Health Organization (WHO) recommends skilled care at every birth and access to facilities with emergency obstetric and neonatal care capacity to prevent maternal and infant deaths (1). Globally, the concept of Maternal Waiting Homes (MWHs) is centred on the pillars of primary health care (PHC) and community health, which includes reproductive, maternal, child, and adolescent health and nutrition. Maternal health strategies embrace Maternal Waiting Homes (MWHs), including the Campaign on Accelerated Reduction of Maternal, Newborn & Child Mortality in Africa (CARMMA) programme in Zambia (2).

Maternal mortality persists as a global health priority on the international development agenda. In Zambia, Maternal Mortality Ratio (MMR) has declined from 591 deaths per 100,000 live births in 2007 to 278 deaths per 100,000 live births in 2018 (3). While 67% of all deliveries are conducted by qualified medical personnel (4). Maternal mortality Ratio (MMR) and maternal complications remain high in Zambia. Pregnant women are affected by many challenges especially in rural areas where hospitals and clinics are not easily accessible as they await delivery, including decision in seeking care, delay in reaching care and delay in receiving adequate care. The construction of Modern Maternal Waiting Homes sort to address these delays, by providing comfortable housing for women as they await delivery and ensure that they deliver at health facilities with specialized health personnel. Modern maternity waiting homes further provide improved maternal and neonatal health in low-risk pregnancies and attach additional emphasis on education and counseling regarding pregnancy, delivery and care of the newborn infant and family. Maternity Waiting Homes (MWHs), also known as mother's shelters, are structures built near healthcare facilities to minimize the critical barrier of distance to accessing maternal health services. They serve as one potential health intervention that may be incorporated into a package of maternal and newborn health services. The Zambian government has identified MMWHs as an intervention to increase demand for maternity care services, improve geographic access to facility delivery especially in rural areas.

Traditionally, communities in Zambia built mud huts to serve as homes for women awaiting delivery. However, utilization was very poor, as women opted to stay in their homes to await delivery due to the poor state of Maternal Waiting Homes (MWHs).Recently, the Ministry of Health (MOH) and cooperating partners have contributed to the construction costs of MMWHs (5) in five provinces in Zambia (Lusaka, Copperbelt, Luapula, Southern and Eastern. Consequently, Maternal Waiting Homes differ in standards and how the women are cared for. The different modes of access are due to the other management mechanisms; MMWHs are supported by the Government and cooperating partners in terms of management and financing, while MWHs are purely community managed. In Zambia, different Cooperating Partners (CPs) have contributed to the construction of MMWHs in other parts of the country, and the facilities and services offered differ. However, utilization of MWHs has been low due to factors such as overcrowding, poor infrastructure, lack of amenities, safety concerns, and cultural issue (6).

The 2030 Sustainable Development Goals (SDGs) aim to ensure healthy lives and promote wellbeing for all people by combining multi-system solutions at global, regional, and national levels, that is, to reduce MMR to 70 maternal deaths per 100,000 live births by 2030 (7). In addressing the challenge of maternal and newborn complications and deaths, the Zambian Government partnered with other stakeholders to construct Modern Maternal Waiting Homes (MMWHs) near health facilities to host pregnant women as a modality to increase safe health facility deliveries. Users are cared for by specialised health personnel (midwives) and bridge the distance barriers from health facilities and communities. Notable strategies include, the National Health Strategic Plan 2017–2021, the Reproductive, Maternal, New-born, Child and Adolescent Health and Nutrition Communication and Advocacy Strategy 2018–2021 (8).

The review of the literature shows that there is little evidence of research on the community voices on factors influencing utilisation of MMWHs in Zambia. Empirical information on the utilisation of MMWHs in Zambia would contribute to the ongoing national efforts by public health practitioners to effectively address the effects of this public health concern. This study therefore aimed to understand community voices on factors influencing utilisation of MMWHs in Zambia.

## Methods

A mixed method study design was employed. The four districts were purposively selected from the provinces where MMWHs had been established in the past four years. Choma district, with a population of 188,693, was selected in Southern Province, Nyimba District, with a population of 101,046 in Eastern Province, Kabwe District, with a population of 234,055 in Central Province and Mansa District in Luapula Province, with a population of 253,414 were selected.

The study was implemented in four districts and four health facilities. Table 1 below indicates the selected districts and provinces.

**Table 1:** The selected districts and provinces in this study.

SN	Province	District	Health facility name	Level of facility
1	Luapula	Mansa	Mutiti health facility	Clinic (Rural health facility)
2	Central	Kabwe	Kabwe mine hospital	Urban Hospital
3	Southern	Choma	Masuku health facility	Clinic (Rural health facility)
4	Eastern	Nyimba	Kacholola health facility	Clinic (Rural health facility)

The study was conducted between January 2021 and December 2022. This study employed qualitative case studies in selected health facilities across Zambia's four districts. Purposeful sampling was used to select study participants using a modern maternal waiting home register as a sampling frame for mothers who had utilised Maternal Waiting Homes and their spouses. Participants were identified using the MWH registers, Safe Motherhood Action Group (SMAG) register and health facility delivery register. The MMWH registers have data on women who had used MMWH. The health facility delivery registers have data on women who delivered from the health facility, maternal and child mortality happening from the health facility, and SMAG registers have data on women who delivered from the community and maternal and child mortality happening in the communities. Selected women were traced with the help of SMAG members situated in the specific zone where these women live.

Sampling of participants through face-to-face, in-depth interviews (IDIs) and focus group discussions (FGDs) was conducted to saturation in all targeted health facilities. Each FGD had separate participants between six and eight (6–8) (women who had utilised MMWHs and spouses to women who had utilised MMWHs in the past year).

Six trained research assistants collected data. They were divided into two groups: three were interviewers, and the other three were recorders and note-takers. Data was collected using three local languages (Bemba, Tonga and Nyanja) spoken in each of the sampled districts, and the research assistants were fluent in the local languages.

The IDIs and FGDs guides were used to solicit information from participants. Both IDIs and FGDs employed a face-to-face interviewing strategy, and all interviews were recorded with the assistance of research assistants. The recorded materials were transcribed with the help of the research assistants. The principal investigator (PI) reviewed and exported the transcribed material for analysis. All study participants were administered information sheets and consent forms. The study was approved by the University of KwaZulu Natal - Health and Social Sciences Research Ethics Centre (HSSREC) reference numbers HSSREC/00002978/2021 and the National Health Research Authority (NHRA) reference number NHRA00014/17/08/2021 and permission was obtained from all District Health Officers (DHO's).

Quantitative data from respondents age variable was performed using Mann-Whitney *U*-test to establish significant difference between the means of two independent groups [IDIs User of MMWH (Females) (*n* = 40)] and FGD (Male partner to users of MMWHs) (*n* = 28). Chi-square tests were applied to establish the association between marital status, education level and employment status the two independent groups [IDIs User of MMWH (Females) (*n* = 40)] and FGD [Male partner to users of MMWHs) (*n* = 28)]. Qualitative data was analyzed using thematic analysis. Summarized data provided a structure in which the researcher systematically reduced the data to tell a story and analyzed it by case and code. Transcribed material was systematically themed. The process of data analysis commenced with data cleaning. The data cleaning process undertook levels of reviews, with the research assistant conducting the first review by listening through the audio and counterchecking the transcribed texts and themes. At the same time, the PI also listened to the recorded audio and compared it with the transcribed material, ensuring that the content was correct. Data was analyzed after each IDI and FDG. It involved reviewing the transcribed material to check for spelling, editing, assigning anchor codes to research questions and matching questions to research questions. The cleaning process also included organizing and keeping track of raw records that were added to the transcripts.

## Results

Table 2 presents the background characteristics of 40 MMWH users and 28 spouses that were interviewed. The largest proportion of MMWH users were aged between 20 and 24 years (25%) and 25 and 29 years (25%). However, there is no statistically significant difference (*p* = 0.6090) between the mean of IDIs User of MMWH (Females) (*n* = 40) and FGD (Male partner to users of MMWHs) (*n* = 28) after conducting a Man Whitney *U*-test. Majority were married (82%) with secondary level education (34%). Nine in 10 of the MMCH (97%) users and spouses were in informal employment (89%).

**Table 2:** Background characteristics of respondents.

Characteristics	IDIs user of MMWH (Females) (*n* = 40)	FGD (Male partner to users of MMWHs) (*n* = 28)
**Age group**		
18–19	5 (12.5%)	5 (18%)
20–24	10 (25%)	5 (18%)
25–29	10 (25%)	5 (18%)
30–34	5 (12.5%)	5 (18%)
35–39	5 (12.5%)	5 (18%)
40+	5 (12.5%)	3 (10%)
Mann-Whitney *U*-test		*p* = 0.6090^a^
**Marital status**		
Single	6 (14%)	2 (6%)
Married	33 (82%)	25 (89%)
Divorced	1 (4%)	1 (5%)
**Level of education**		
No education	12 (29%)	6 (22%)
Primary	13 (33%)	8 (27%)
Secondary	14 (34%)	11 (41%)
Tertiary	1 (4%)	3 (10%)
**Employment status**		
Informal employment	39 (97%)	25 (89%)
Formal employment	1 (3%)	3 (11%)

^a^ Mann-Whitney test: *P*-value score = 0.6090. This shows that here is no statistically significant difference (*p* = 0.6090) between the mean of IDIs User of MMWH (females) (*n* = 40) and FGD (Male partner to users of MMWHs) (*n* = 28).

Table 3 shows the relationship between background characteristics and the two independent groups: IDIs User of MMWH (Females) and FDG Male partner to users of MMWHs. Results show that there were no significant differences between the respondents with User of MMWH (Females) and Male partner to users of MMWHs regarding marital status (*p* = 0.756), educational level (0.157) and employment status (0.157).

**Table 3:** Percent distribution of sex by background characteristics.

Background characteristics	User of MMWH	Partner to users of MMWHs	*P*-value
**Marital status**			
Never married	1 (50%)	1 (50%)	*p* = 0.756^a^
Married	34 (57.6%)	25 (42.4%)	
Divorced	5 (71.4%)	2 (28.6%)	
**Education status**			
No education	12 (66.7%)	6 (33.3%)	*p* = 0.157^b^
Primary	13 (61.9%)	8 (38.1%)	
Secondary	14 (56.0%)	11 (44.0%)	
Tertiary	1 (25.0%)	3 (75.0%)	
**Employment status**			
Formal employment	1 (25.0%)	3 (75%)	*p* = 0.157^c^
Informal employment	39 (60.9%)	25 (39.1%)	

^a^ Mann-Whitney test: The *P*-value shows that there were no significant differences between the respondents with User of MMWH (Females) and Male partner to users of MMWHs regarding marital status (*p* = 0.756).

^b^ Mann-Whitney test: The *P*-value shows that there were no significant differences between the respondents with User of MMWH (Females) and Male partner to users of MMWHs regarding educational level (*p* = 0.157).

^c^ Mann-Whitney test: The *P*-value shows that there were no significant differences between the respondents with User of MMWH (Females) and Male partner to users of MMWHs regarding employment status (*p* = 0.157).

## Delivering from MMWH

Users provided beneficial information on the use of MMWHs and highlighted the history of using the MMWHs. Below are some selected quotes;

“*………I have used the MMWH, I used it on my first delivery in 2019. I recall, I found about four women in the shelter. I stayed there for about two weeks and three other women joined. The place had just been built and was perfect*” [User 1, Mutiti health centre - Mansa]

“*……I stayed at the mothers shelter. I found six other women. I was the youngest on the group and all the women took me as their young sister. I stayed there for about 10 days and by the time i was leaving, i left two women, all others had delivered and left*” [User 2, Kacholola health centre - Nyimba]

## Reasons for utilising the MMWHs

The primary reasons for utilising MMWHs were the comfortable state of MMWH, the long distance from home to the health facility, fear of maternal complications, availability of specialised health personnel, and the information on the benefits of utilising the MMWH necessitated them to use the waiting home.

Most of the women who had utilised the MMWHs were living far from the health facilities and felt the waiting homes were comfortable; therefore, it was very beneficial for them to deliver at the health facility. It was easier for the women to travel long distances to the health facility a week or two before delivery than to be moved using unreliable modes of transport hours before delivery. The primary mode of transport in most rural Zambia is through bicycles and sometimes wheelbarrows for women awaiting delivery, as most rural parts of the country have poor road networks to facilitate vehicle passage.

Further, no maternal and newborn complications, or deaths over the study period across all health facilities. This gave confidence to would be users to use the MMWH's. Some women highlighted the following as the reasons for using the MMWHs;

“*…..the modern maternal waiting homes are very comfortable, they are clean and the furniture is also good. It doesn't show much difference with being home*” User 6, [Kabwe Mine hospital - Kabwe]

“*…………..I had a bleeding problem, so I thought it would be best to move closer to the hospital. I also stay very far and fear of delivering in the village and the problems it brings*” User 3, Mutiti health facility - Mansa}

Some users indicated that they had used the MMWH because of the many maternal complications they experienced. The consequences were terrible, as some recounted the loss of life as a result of the complications due to the delay in accessing care when complications occurred at home with no specialised personnel readily available. Most communities had experienced deaths as a result of these, and it gave them the primary reason to seek medical care during delivery for their safety and that of their newborns.

“*…….my niece died on her way to the clinic, labour had started and she lost a lot of blood. The woman who used to attend to mothers in the community tried but the situation was worsening. She died because there were no specialised people to attend to her, had she been at the health centre, she could not have died*” [User 9, Masuku health facility - Choma]

Some women resorted to utilising the MMWHs because they had gotten beneficial information about the waiting homes during ANC. At every ANC visit, nurses encouraged pregnant women to stay at the waiting home before delivery, as the health workers would often check on the women and ensure they were attended to promptly or referred to another level of care. Women waiting at the MMWH were also taught other skills. The skills were aimed at equipping women with practical income-generating activities (IGAs) that would enable them to provide extra income for their households and broaden their businesses. These included sewing (as all MMWHs had sewing machines), keeping fit activities, goat rearing, business management and hammer mill management. Other benefits included easily accessible medical services.

“*…….the health care I was given at the clinic during pregnancy, especially even during my first delivery was reason enough to use the waiting home after my second delivery*” [User 10, Mine Health Facility - Kabwe]

## Days spent at the MMWHs

Length of stay was based on individual needs and contexts. Most respondents said they spent an average of two weeks at the waiting home. Some spent over a month awaiting delivery. This occurred due to complications identified earlier in pregnancy.

“*….had been in the waiting home for fourteen (14) days. During my stay, there were five (5) other women who were in the waiting home and stayed around 14 days also, though others left quite earlier*” [User 12, Kacholola health facility - Nyimba]

## Perceptions of MMWHs

Users were' positive about their experiences at the MMWHs.' Women who stay at home during their entire pregnancy risk complications during the latter stage of pregnancy and during labour.

“*……….It was easy for me to deliver because when my time came, I just went straight into emergency care without waiting. I also observed that, in instances where people arrive late in the night, they have to struggle to get a nurse or birth attendant on site quickly, so it's a challenge in emergency situations*” [User 14, Mutiti Health Facility - Mansa]

## Spouse's views on the MMWHs

Spouses were asked about their views on the MMWHs in FGDs. Spouses suggested differences in the two groups of mothers (users and non-users). Women who delivered at home (non-users) experienced difficulties in giving birth because they were not monitored by the health workers and delivered in unsafe environments. Those who utilised the MMWH were closely monitored by nurses in case of any emergency and had modern equipment and furniture.

“*…………..If one shifts at the maternal waiting home, there is fast care, but those coming from home face a lot of problems especially in movement during labour which cause deaths and also the places of delivery are unsafe. For example if I am at the maternal waiting home, I will be helped fast if I face any problem and if my child faces any problem*” [FGD-User spouses 4, Masuku Health Facility - Choma].

“*……..this maternal waiting home is very good, it is an attraction to most women to come and stay at, as they await delivery. It has nice furniture in terms of beds, cooking facilities and other amenities such as sewing machine lessons that women tend to benefit from whilst staying at the maternal waiting home*” [FGD-User Spouse 6, Mutiti health facility - Mansa]

## Challenges faced at the MMWH

Staying at the MMWH also presents various challenges for pregnant women. The main challenges women face include financial resources, food for upkeep, charcoal and firewood and limited help from relatives, as there are restrictions on the number of relatives who could accompany the awaiting mother. Some respondents indicated that;

“*…..Staying at the MMWH is absolutely free, however they don't provide any food staff or cooking material. if your relatives don't bring food, some money and other necessities, you could get stranded*” [User 15, Mine Health Facility - Kabwe]

Participants further indicated that their stay at the waiting home affected the financial burden on their families. Families had to share the food and other necessities between home and the MMWH requirements. The quote below illustrates this point;

“*……..it's obvious that my household's financial standpoint was affected by my stay at the MMWH. This is because they had to divide the food and money for home. At the waiting home, I needed some money for upkeep. I bought vegetables, fish and cooking oil (foodstuff) and washing paste and other cleaning materials*” [User 16, Mutiti Health Facility - Mansa]

### Attitudes and behaviours of the health personnel at the MMWHs

Participants indicated that health workers at the hospital gave them the required attention and care during their stay at the shelter. They added that the nurses' attitudes were good because they were very supportive and encouraging to them. This is evidenced below:

“*….it was encouraging for me to stay at the MMWH. Health workers were very caring, supportive and encouraging. I would encourage my fellow women to use the maternal waiting home in their next delivery*” [User 18, Mine Health Facility - Kabwe]

The FGD participants were asked if health workers at the hospital gave their spouses the required attention and care during their stay at the MMWH. The health workers cared for and encouraged participants whilst at the MMWH. Nurses' attitudes were good as they were very supportive and encouraging to awaiting mothers. responses from spouses:

“*My spouse (wife) said that the nurses and all staff at the health centre were supportive and very friendly to women.”* [FGD-User Spouse 8, Kacholola Health Facility - Nyimba]

## Views of spouses regarding women using an MMWH

The study reviewed spouses' views on their spouses utilising the MMWH as they awaited delivery. The spouse's position on the utilisation of health services has a bearing on the financial resources that must be shared and on the domestic chores usually performed by the women in local homes. Most pregnant women indicated that husbands/spouses were supportive of them utilising the MMWH. Some respondents indicated the following;

“*..my husband was okay with me staying two weeks at the waiting home……. he had no problem with it*” [User 19, Mine Health Facility - Kabwe]

“*…..He was okay with it; he thought it was a good idea to stay there, and I am sure he would encourage me to go there again.*” [User 20, Masuku Health Facility - Choma]

## Effects of the MMWH on improving health outcome of the mother and child after birth

Mothers who utilised the MMWH indicated that mothers who stay at the MMWH have more benefits than their colleagues who have not used the waiting homes because of the information and teachings the users are privileged to receive. Among them are teachings on postnatal care (PNC) and other health services given to them by health workers. One response indicated that;

“*…..when you are at the MMWH, there are many lessons that are taught. There subjects on postnatal care that are important for the mother, as they form a strong base for child survival*” [User 21, Mutiti Health facility - Mansa]

The study assessed the possible effects of mothers using the MMWH on mortality. Participants thought that not using MMWH contributes to women having maternal and child mortality, as women who do not utilise the MMWH face the risks. Below are some views from women users;

“*Yes, women who do not use the MMW have risks, especially when they get into labour; they could be at risk of a breech, and there would be no medical assistance nearby*” [User 22, Kacholola Health Facility - Nyimba]

## Discussion

It is imperative to note that knowledge and awareness of MMWHs are almost universal across all districts and health facilities. However, they are not reflective of use, as a substantive number of women are still not using MMWHs despite them being upgraded and modernised. This is consistent with a Ghanaian study by Konlan (9) that argued that low utilisation of maternal health services is not due to a lack of knowledge about the benefits of maternal healthcare use. Instead, other factors might be influencing pregnant women's health-seeking behaviour (9). This finding suggests that knowledge is a necessary pre-requisite in adopting health-promoting behaviours, but it may not be sufficient to enable behaviour change. The women in the referenced communities were aware of the improvements made to the newly constructed MMWHs and knew about health personnel in attendance.

Notably, in all four health facilities across the four districts, all women who utilised MMWHs delivered at health facilities. Our study indicates that most spouses supported their partners in utilising MMWHs because of their perceived benefits the study further established that there was consensus among couples in deciding on utilising the waiting. Our findings are consistent with another study by Sialubanje (10), who argued that women with decision-making autonomy in a household were more likely to influence their own utilisation of the MMWH compared to women who did not have decision-making autonomy (11). Vermeiden (12) argued that women with decision-making autonomy used the MWH better than those without. difference in MMWHs utilisation between women with no education and those with primary education was minimal.

Perceptions of users of MMWH indicates improved care by health providers, available health providers and equipment, avoiding complications, improved waiting homes and easy referrals to advanced care. Findings are consistent with Tayebwa (13) who established that MWH offered a peaceful and home-like environment, good-quality services, or timely obstetric services, and was associated with good maternal and neonatal outcomes. Molla (14) argued that, in Ethiopia, mother's perception of maternity waiting homes highlighted low adverse maternal and perinatal birth outcomes compared to non-users.

This study indicated that not using MMWH might contribute to women having maternal and child mortality. Findings resonate with Molla (14) who argues that women who stay in the MWH before giving birth have a substantial impact on lowering unfavourable maternal and perinatal birth outcomes compared to mothers who did not stay in the maternity waiting home. Our findings further demonstrate that women who do not utilise MMWH are likely to encounter maternal risks and are devoid of benefits such as teachings on postnatal care (PNC) and other life skills.

Our study found that health workers at various facilities provided their clients with the required attention and care during their stay at the MMWH. The attitudes of providers were good, supportive and encouraging. On the contrary, a systematic review by Mannava (15) indicated that the majority (55/81) of studies outlined negative attitudes or behaviours of providers. Overall, negative attitudes and behaviours undermine healthcare-seeking and affect patient well-being. On the contrary, Kapesa (16) found that maternal waiting home users stated that healthcare workers had bad attitudes towards clients, further, some providers requested pregnant women do their work.

The fear of experiencing maternal complications whilst in communities with no healthcare personnel to attend to expecting mothers made most women prefer to utilise the MMWH and subsequently deliver from the health facility. This is consistent with Sialubanje's (17) 2015 findings in Kalomo Zambia, where respondents who had used MWHs and those who had experienced complications during previous pregnancies mentioned the risk of complications as the major reason for utilising MWHs (17). Communities that had experienced maternal deaths reinforced the primary reason to seek medical care during delivery. Being attended to by the midwives at the health facility was another contributor to most participants using the MMWHs. Findings further indicated that some mothers utilised the MMWH because they lived far from health facilities. The women who stayed longer distances from the health facilities travelled and stayed at the MMWH a week or two before delivery. Similarly, Sialubanje (17) indicated that distance from pregnant women's home to health facilities was an important factor that the families considered during the decision-making process is distance (18).

Our study indicates that staying at the MMWH presents various challenges for pregnant mothers. Among the recorded challenges are financial resources, food for upkeep, charcoal and firewood, and limited help from relatives. Our findings are consistent with Gurara (19), who argued that in Ethiopia, users of MWHs experienced challenges relating to distance, transportation, financial costs (higher out-of-pocket payments), and poor provider interactions with women staying at MWHs.

## Conclusion

MMWHs are a pivotal intervention in improving maternal outcomes. All health facilities had no maternal and newborn complications or deaths over the study period. It is also cardinal to note that MMWH are equipped with life-saving skills that users are trained in as they await delivery and further benefit from the special checkups by health personnel. It is, however, notable that users of MMWHs also experience some financial constraints to support their stay at the MMWH, as there are standards of what each user is expected to have. However, these don't outweigh the risks. Spouses of users play a cardinal role in supporting their spouses and encouraging them to use the MMWH.

## Data Availability

The raw data supporting the conclusions of this article will be made available by the authors, without undue reservation.
